# Efficacy and safety of endovascular coiling compared to microsurgical clipping in aneurysmal subarachnoid hemorrhage: a systematic review and meta-analysis of randomized trials

**DOI:** 10.1007/s00415-026-14150-z

**Published:** 2026-10-08

**Authors:** Martin Vychopen, Karl-Titus Hoffmann, Erdem Güresir, Johannes Wach

**Affiliations:** 1 Department of Neurosurgery, University Hospital Leipzig, Liebigstraße 20, 04103 Leipzig, Germany; 2 Department of Neuroradiology, University Hospital Leipzig, Leipzig, Germany

**Keywords:** Subarachnoid hemorrhage, Intracranial aneurysm, Endovascular coiling, Surgical clipping, Meta-analysis

## Abstract

**Background:**

Aneurysmal subarachnoid hemorrhage (aSAH) carries high morbidity and mortality despite advances in aneurysm repair. Microsurgical clipping and endovascular coiling are established treatment modalities, yet their relative benefits regarding functional outcome and long-term aneurysm occlusion remain debated. This study evaluates treatment effects stratified by aneurysm location and clinical grade.

**Methods:**

We identified RCTs comparing clipping and coiling for ruptured intracranial aneurysms. Primary outcomes were favorable functional outcome at 1-year [modified Rankin Scale (mRS), and complete aneurysm occlusion at 1-year]. Secondary outcomes included location-specific analyses, WFNS grade-specific analyses, and all-cause mortality.

**Results:**

Six RCTs involving up to 3044 patients were included. Coiling was associated with higher odds of favorable outcome at 1 year using mRS 0–2 (OR 1.60, 95% CI 1.36–1.88; *p* < 0.00001), but not when using mRS 0–3 (OR 1.19, *p* = 0.12). Location-specific analyses showed better outcomes after coiling for internal carotid artery aneurysms (OR 0.44, *p* < 0.00001), no difference for anterior communicating, anterior cerebral, middle cerebral, or posterior circulation aneurysms. Complete occlusion at 12 months favored clipping overall (OR 0.35, 95% CI 0.28–0.44; *p* < 0.00001; *I*^2^ = 0%), driven by superiority in middle cerebral artery aneurysms (OR 5.21, *p* = 0.007). Mortality did not differ (OR 0.89, *p* = 0.35).

**Conclusions:**

Coiling offers superior 1-year functional outcomes when assessed with mRS 0–2, while clipping achieves higher complete occlusion rates, predominantly in middle cerebral artery aneurysms. No mortality difference was found.

**Supplementary Information:**

The online version contains supplementary material available at https://doi.org/10.1007/s00415-026-14150-z.

## Introduction

Aneurysmal subarachnoid hemorrhage (aSAH) remains a devastating neurological emergency with an estimated incidence of 6–9 cases per 100,000 person-years and a case fatality rate reaching 35%, despite advancements in aneurysm repair and neurocritical care management [1]. While early aneurysm occlusion remains the mainstay of treatment to prevent rebleeding, the optimal technique—microsurgical clipping or endovascular coiling—continues to be debated [2]. Endovascular coiling, following the landmark ISAT-1 trial, has become the preferred modality in many centers, primarily for patients with anterior circulation aneurysms and good clinical grades [3].

However, concerns persist regarding the long-term durability of coiling and its lower rates of complete aneurysm occlusion compared to clipping [4]. Most recently, the ISAT-2 trial by Darsaut et al. [5] expanded the evidence base by randomizing patients previously excluded from earlier trials, providing more generalizable insights into the comparative outcomes of coiling and clipping in broader clinical scenarios.

Current guideline recommendations emphasize an individualized approach to aneurysm treatment, incorporating patient age, aneurysm location, and clinical status [6]. Despite the availability of multiple meta-analyses comparing both techniques, most have focused on aggregated endpoints without stratification for crucial factors such as aneurysm site or baseline aSAH severity [7]. This has led to a knowledge gap regarding the role of aneurysm location in influencing functional outcome and is currently predominant based on the subgroup analyses from ISAT-1 [8].

Furthermore, complete aneurysm occlusion—a critical parameter to prevent rebleeding—has rarely been evaluated systematically across randomized trials in a location-specific manner. Similarly, functional outcomes such as the modified Rankin Scale (mRS) are often pooled across heterogeneous subgroups, limiting insight into anatomically stratified benefit [9].

A recent meta-analysis of individual patient data demonstrated that complete obliteration of an aneurysm prevents potentially disastrous rebleeding events [7]. Furthermore, according to the Barrow Ruptured Aneurysm Trial [10], complete clip-occlusion exhibits superior treatment durability, resulting in a significantly lower necessity for retreatment. Notably, both endovascular and microsurgical retreatment procedures are associated with elevated perioperative complication rates compared to primary interventions [11]. Consequently, achieving complete obliteration in a single procedure remains one of the primary therapeutic goals. A treatment stratification according to anatomical localization might further optimize the therapeutic strategy for each ruptured aneurysm.

Therefore, in the present meta-analysis of randomized controlled trials (RCTs), we aim to address these limitations by conducting a stratified comparison of coiling versus clipping.

## Methods

This meta-analysis was conducted in accordance with the Preferred Reporting Items for Systematic Reviews and Meta-Analyses (PRISMA) guidelines. The study protocol was registered in the PROSPERO database (Registration number: CRD420251158098).

A comprehensive literature search was performed across PubMed, Embase, and Cochrane Library. Search terms included combinations of keywords and Medical Subject Headings (MeSH) related to “aneurysm,” “clipping,” “coiling,” “endovascular treatment,” and “randomized controlled trial”. Reference lists of relevant articles and previously conducted meta-analyses were manually screened to identify additional eligible studies.

Two reviewers independently screened titles, abstracts, and full texts to identify randomized controlled trials comparing endovascular coiling versus surgical clipping for ruptured intracranial aneurysms. Data extraction was independently performed by the same reviewers. Discrepancies regarding study inclusion were resolved by a third and fourth author.

### Search strategy

We searched for all clinical studies published between 1995 and 2025. MESH Terms included “subarachnoid hemorrhage”, “aneurysm”, “coiling”, “clipping”. Excluded were letters, opinions, narrative reviews, case reports and study protocols. Subsequently, we excluded single-arm studies, studies with missing report on primary and secondary outcomes and studies on unruptured aneurysms.

For functional outcome analysis using modified Rankin Scale (mRS) 0–2 vs. mRS 3–6, 6 studies were included (Koivisto et al. [12], Molyneux et al. [3], Li et al. [13], McDougal et al. [14], Wadd et al. [15], Darsaut et al. [5]). For functional outcome analysis using mRS 0–3 vs. mRS 4–6, 3 studies were included (Koivisto et al. [12], Molyneux et al. [3], Darsaut et al. [5]). Outcome analysis based on aneurysm localization and on WFNS grade was performed using two studies (Molyneux et al. [3], Darsaut et al. [5]). Mortality analysis was conducted using six studies (Koivisto et al. [12], Molyneux et al. [3], Li et al. [13], McDougal et al. [14], Wadd et al. [15], Darsaut et al. [5]). Complete occlusion analysis was performed with four studies (Koivisto et al. [12], Molyneux et al. [3], Li et al. [13], Darsaut et al. [5]), including two studies evaluating occlusion rates based on aneurysm location (Koivisto et al. [12], Darsaut et al. [5]). For details on the search strategy, see Supplementary Fig. 1.

### Statistics

Statistical analyses were performed using Review Manager (RevMan, version 5.4.1, Cochrane Collaboration). For each study, odds ratios (ORs) with corresponding 95% confidence intervals (CIs) were calculated for dichotomized clinical outcomes based on the modified Rankin Scale (mRS). A random-effects model was applied to account for potential heterogeneity between included studies. Heterogeneity was quantified using the *I*^2^ statistic, with values of 25%, 50%, and 75% representing low, moderate, and high heterogeneity.

Forest plots were generated in RevMan to visually present study-specific and pooled ORs. Funnel plots were constructed to assess the potential for publication bias.

### Risk of bias assessment

The methodological quality of the included studies was assessed using the Cochrane Risk of Bias 2.0 tool. Any differences in risk of bias assessments were resolved by consensus [16]. The certainty of the evidence was appraised following the Grading of Recommendations Assessment, Development, and Evaluation (GRADE) framework.

## Results

### Included studies

The included RCTs spanned multiple regions, enrolling between 109 and 2143 patients. Most studies were conducted in single centers except ISAT [3], which was multinational with 42 centers. Across trials, clipping and coiling were balanced, with coiling slightly favored in larger studies. Severity grading was commonly reported using the WFNS scale; the proportion of low-grade (WFNS 1–2 or H&H I–III) patients ranged from 64% (BRAT) [14] to 88% (ISAT) [3], with Wadd et al. [15] exclusively enrolling WFNS grades 1–3. Aneurysm locations were predominantly in the anterior circulation. ACA aneurysms were the most common across studies (33–45%), followed by MCA (25–38%) and ICA (17–30%). Posterior circulation aneurysms comprised up to 18% in some studies but were excluded in Wadd et al. [15]. Most aneurysms were small, with ≥ 85% less than 10–12 mm in ISAT [3], BRAT [15], and Li et al. [13]. These consistent patterns in aneurysm type and severity support comparability across trials in the meta-analysis.

### Outcome and mortality

#### Cumulative outcome (mRS 0–2 vs. mRS 3–6)

Six RCTs comparing endovascular coiling versus surgical clipping for intracranial aneurysm treatment were included for this analysis. The available data were pooled as favorable clinical outcome (mRS 0–2) versus unfavorable outcome (mRS 3–6). The resulting cohort included 3044 patients (1431 coiling, 1613 clipping).

**Fig. 1:**
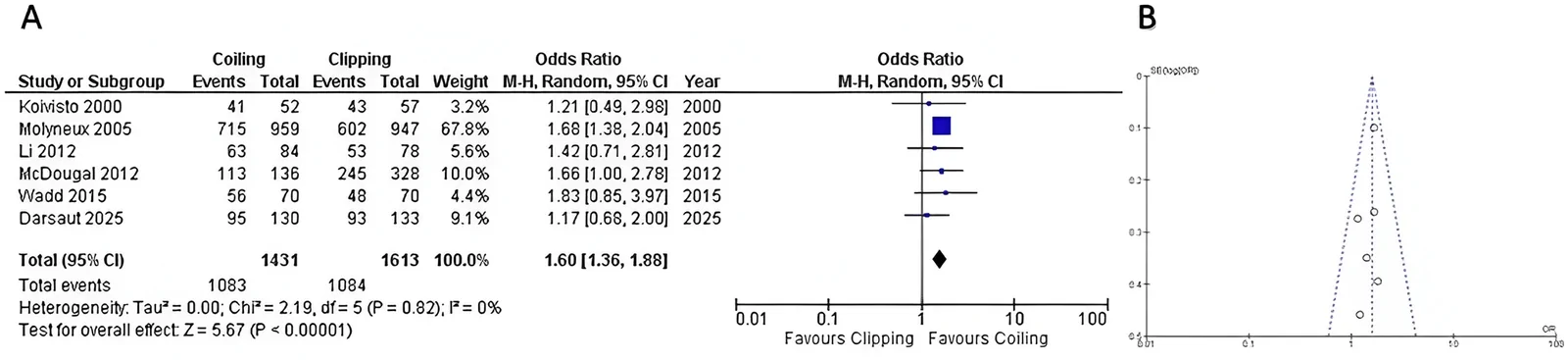
**A** Forest plot displaying odds ratio (OR) and 95% confidence interval (CI) estimates for favorable clinical outcome (mRS 0–2) from randomized controlled trials comparing coiling versus clipping in patients with intracranial aneurysms. Squares represent individual study ORs, with square size proportional to the study’s weight (inversely related to CI width). The diamond represents the pooled OR, demonstrating the overall effect estimate from all included studies. **B** Funnel plot assessing publication bias. The distribution of included studies is symmetrical, indicating no relevant publication bias

The cumulative odds ratio (OR) for favorable outcome significantly favored coiling over clipping (OR 1.60; 95% CI 1.36–1.88; *p* < 0.00001) using a random-effects model. Heterogeneity was low (*I*^2^ = 0%), indicating consistency among studies. Individual study ORs ranged from 1.17 (Darsaut et al.) [5] to 1.86 (McDougal et al.) [14]. The largest contribution to the pooled effect was from ISAT study (Molyneux et al.) [3] with weight of 67.8% (Fig. 1A).

The Funnel plot demonstrates symmetrical distribution around the pooled OR, indicating no substantial evidence of publication bias (Fig. 1B).

#### Cumulative outcome (mRS 0–3 vs. mRS 4–6)

Three RCTs provided data for analyzing the outcome based on different dichotomization cut-off (favorable outcome defined as mRS 0–3 vs. unfavorable outcome defined as mRS 4–6). The resulting cohort included 2278 patients (1141 coiling, 1137 clipping).

The cumulative odds ratio (OR) for favorable outcome showed no statistically significant difference between coiling and clipping (OR 1.19; 95% CI 0.96–1.48; *p* = 0.12) using a random-effects model. Heterogeneity was low (*I*^2^ = 0%), indicating strong consistency across the included studies. Individual study ORs ranged from 1.02 (Darsaut et al.) [5] to 1.22 (Molyneux et al.) [3], with the ISAT study (Molyneux et al.) [3] contributing the largest weight (81.9%) to the pooled estimate. For detailed information, refer to Supplementary Fig. 2.

#### Outcome (mRS 0–2 vs 3–6) based on aneurysm location

Two RCTs provided data for analyzing the outcome based on location of the aneurysm. The outcome was defined as favorable (mRS 0–2) and unfavorable (mRS 3–6).

**Fig. 2:**
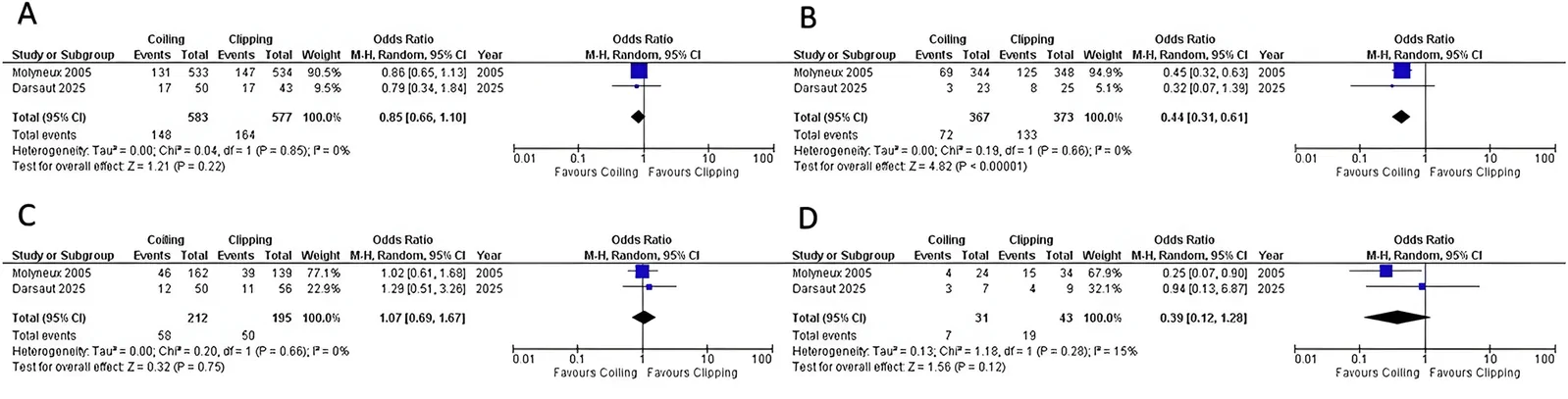
**A** ACA and A-com aneurysms: Forest plot displaying odds ratio (OR) and 95% confidence interval (CI) estimates for favorable clinical outcome (mRS 0–2). **B** ICA aneurysms: Forest plot displaying odds ratio (OR) and 95% confidence interval (CI) estimates for favorable clinical outcome (mRS 0–2). **C** MCA aneurysms: Forest plot displaying odds ratio (OR) and 95% confidence interval (CI) estimates for favorable clinical outcome (mRS 0–2). **D** Posterior circulation aneurysms: Forest plot displaying odds ratio (OR) and 95% confidence interval (CI) estimates for favorable clinical outcome (mRS 0–2). **A**–**D** Squares indicate study-specific ORs, with square size reflecting study weight. The diamond represents the pooled OR from the random-effects model

Based on the pooled data, we saw no significant difference in outcomes between coiling and clipping in ACA and A-com aneurysms (Fig. 2A; *p* = 0.22), MCA aneurysms (Fig. 2C; *p* = 0.75) and posterior circulation aneurysms (Fig. 2D; *p* = 0.12).

Patients with ICA Aneurysms demonstrated statistically significant difference between both treatment modalities. Cumulative odds ratio (OR) for favorable outcome showed significant difference in favor of coiling (Fig. 2B, OR 0.44; 95% CI 0.31–0.61; *p* = 0.00001) using a random-effects model. Heterogeneity was low (*I*^2^ = 0%).

#### Outcome based on WFNS Grade

##### WFNS ≤ 3

We stratified the neurological outcome dichotomized as favorable (mRS 0–2) and unfavorable (mRS 3–6) according to WFNS Grade of the patients. 2 RCTs met the inclusion criteria.

We identified 2213 patients available for the analysis. 1106 underwent coiling and 1107 underwent clipping. Outcome by patients with WFNS ≤ 3 was significantly better if patients underwent coiling (OR 0.65; 95% CI 0.54–0.79; *p* = 0.0001). The heterogeneity of the studies was low (*I*^2^ = 0%). For detailed information, refer to Supplemental Fig. 3.

##### WFNS4 > 3

We identified 168 patients available for the analysis. 87 underwent coiling and 81 underwent clipping. Outcome by patients with WFNS > 3 did not show any statistical significance (OR 1.12; 95% CI 0.60–2.10; *p* = 0.72). The heterogeneity of the studies was low (*I*^2^ = 0%).

For detailed information, refer to Supplemental Fig. 4.

#### Mortality

Six RCTs provided data for analyzing mortality. The resulting cohort included 2921 patients (1463 coiling, 1458 clipping).

The cumulative odds ratio (OR) showed no statistically significant difference between coiling and clipping (OR 0.89; 95% CI 0.69–1.14; *p* = 0.35). Heterogeneity was low (*I*^2^ = 0%), indicating strong consistency across the included studies. Individual study ORs ranged from 0.32 (Wadd et al.) [15] to 1.33 (Darsaut et al.) [5], with the ISAT study (Molyneux et al.) [3] contributing the largest weight (57.2%) to the pooled estimate.

For detailed information, refer to Supplemental Fig. 5.

### Complete occlusion

#### Complete occlusion in 12 months: cumulative results

Four RCTs provided data for analyzing complete occlusion of the aneurysm 12 months after initial treatment. The resulting cohort included 1889 patients (1157 coiling, 732 clipping).

**Fig. 3:**
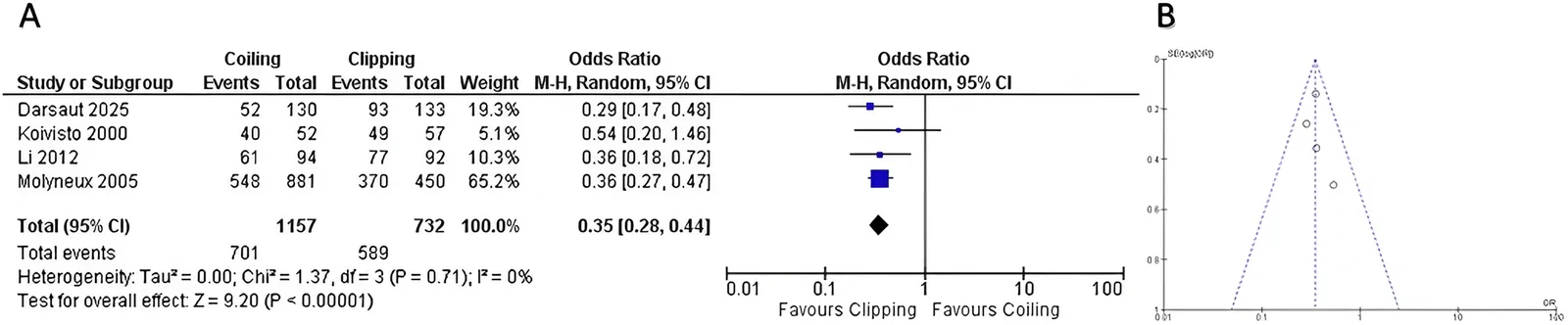
**A** Forest plot displaying odds ratio (OR) and 95% confidence interval (CI) estimates for complete occlusion from randomized controlled trials comparing coiling versus clipping in patients with intracranial aneurysms. Squares represent individual study ORs, with square size proportional to the study’s weight (inversely related to CI width). The diamond represents the pooled OR, demonstrating the overall effect estimate from all included studies. **B** Funnel plot assessing publication bias. The distribution of included studies is symmetrical, indicating no relevant publication bias

The cumulative odds ratio (OR) showed statistically significant difference in favor of clipping (OR 0.35; 95% CI 0.28–0.44; *p* = 0.00001). Heterogeneity was low (*I*^2^ = 0%). Individual study ORs ranged from 0.29 (Darsaut et al.) [5] to 0.54 (Koivisto et al.) [12], with the ISAT study (Molyneux et al.) [3] contributing the largest weight (65.2%) to the pooled estimate (Fig. 3).

#### Complete occlusion in 12 months: stratification based on aneurysm location

Two RCTs provided data for analyzing the complete occlusion based on location of the aneurysm. Complete occlusion had to be confirmed in angiography performed 12 months after initial treatment.

**Fig. 4:**
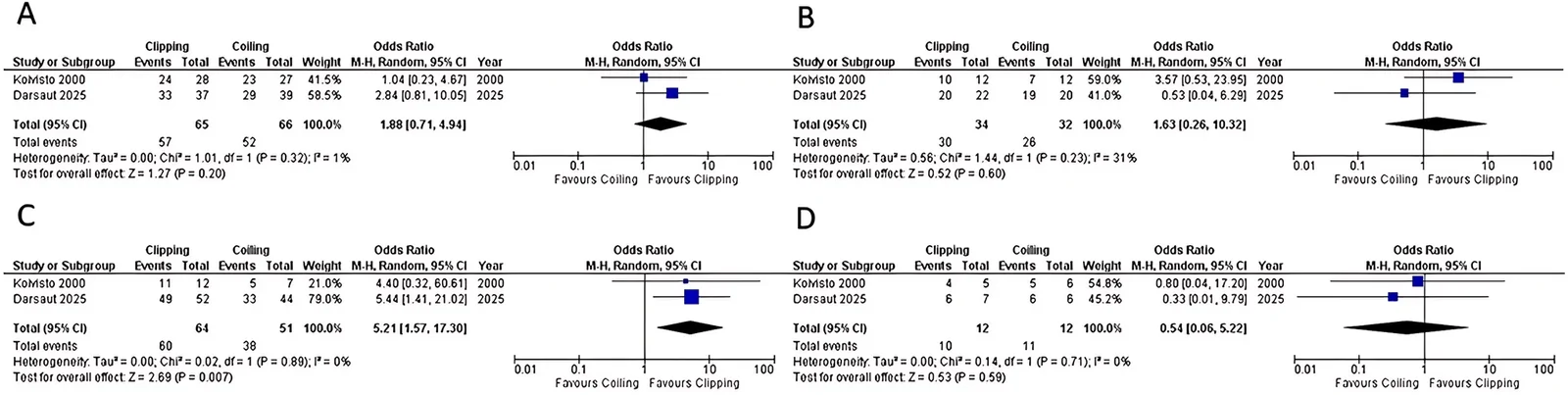
**A** ACA and A-com aneurysms: Forest plot displaying odds ratio (OR) and 95% confidence interval (CI) estimates complete occlusion in 12 months. **B** ICA aneurysms: Forest plot displaying odds ratio (OR) and 95% confidence interval (CI) estimates for complete occlusion. **C** MCA aneurysms: Forest plot displaying odds ratio (OR) and 95% confidence interval (CI) estimates for complete occlusion. **D** Posterior circulation aneurysms: Forest plot displaying odds ratio (OR) and 95% confidence interval (CI) estimates for complete occlusion. **A**–**D** Squares indicate study-specific ORs, with square size reflecting study weight. The diamond represents the pooled OR from the random-effects model

Based on the pooled data, we saw no significant difference in complete occlusion in 12 months between coiling and clipping in following localizations: ACA and A-com aneurysms (Fig. 4A; *p* = 0.20), ICA Aneurysms (Fig. 4B; *p* = 0.60) and posterior circulation aneurysms (Fig. 4D; *p* = 0.59).

Patients with MCA Aneurysms demonstrated statistically significant difference between both treatment modalities. Cumulative odds ratio (OR) for complete occlusion showed significant difference in favor of clipping (OR 5.21; 95% CI 1.57–17.30; *p* = 0.007, Fig. 4C) using a random-effects model. Heterogeneity was low (*I*^2^ = 0%).

### Risk of bias

**Fig. 5:**
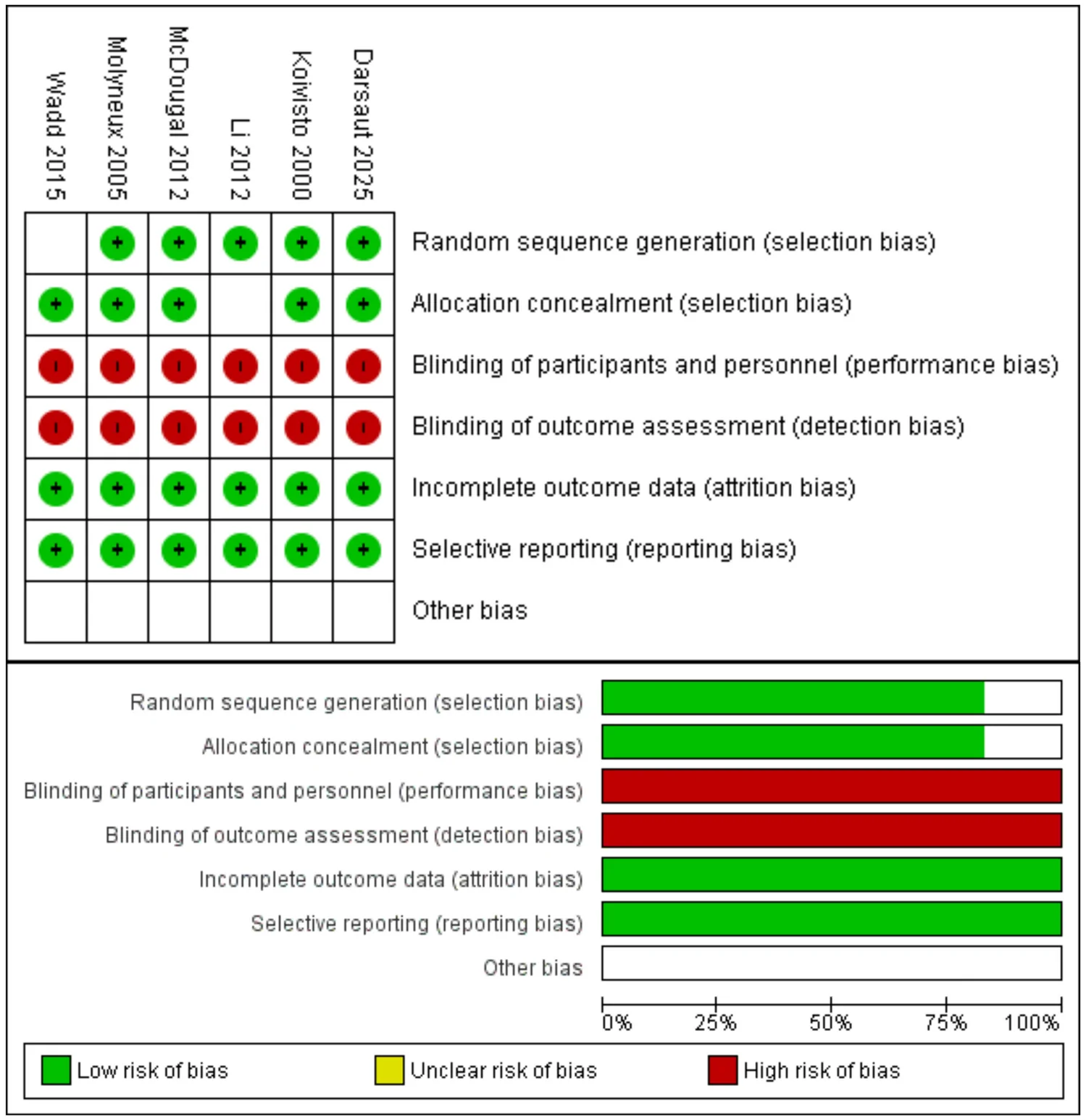
Risk of bias assessment for included randomized controlled trials Each domain is color-coded as low risk (green), some concerns (yellow), or high risk (red) across the five bias domains: randomization process, deviations from intended interventions, missing outcome data, measurement of outcome, and selection of the reported result

The risk of bias assessment revealed that most studies demonstrated generally acceptable methodological quality, although concerns were noted in specific domains. Darsaut et al. [5] showed some concerns due to missing outcome data, as a subset of randomized patients was excluded without sufficient justification. Koivisto et al. [12] lacked clarity in randomization procedures and had incomplete neuropsychological follow-up, contributing to concerns across multiple domains. Li et al. [13] raised issues due to unclear allocation methods and absence of a preregistered protocol, suggesting potential selective reporting. McDougall et al. [14] (BRAT) and Molyneux et al. [3] (ISAT) were assessed as low risk across all domains due to rigorous design, transparent reporting, and robust follow-up procedures. In contrast, Wadd et al. [15] had some concerns in randomization and missing data and a high risk for selective reporting due to lack of protocol transparency and unclear outcome definitions. These findings highlight variability in methodological rigor among included RCTs (Fig. 5).

## Discussion

In this meta-analysis of randomized controlled trials on aneurysmal subarachnoid hemorrhage, we found that coiling was associated with higher odds of favorable outcome (mRS 0–2) at 1 year, although this benefit was not observed when using the broader mRS 0–3 definition. Mortality did not differ between modalities. Location-specific analyses showed a significant advantage for coiling in internal carotid artery aneurysms, whereas clipping achieved superior complete occlusion rates in middle cerebral artery aneurysms. These findings underscore the need for individualized treatment selection based on aneurysm location and patient-specific factors.

In the ISAT-1 trial (Molyneux et al. [3]), the reported benefit of endovascular coiling over surgical clipping at 1 year was based on dichotomization of the modified Rankin Scale (mRS) into 0–2 (favorable) vs. 3–6 (unfavorable). However, Ausman et al. [17] and Sade et al. [18] critically pointed out that this distinction is not differentiated enough and sensitive to how the mRS is grouped. Our pooled data including the recent ISAT-2 [5] study further supports this by demonstrating that when outcomes are dichotomized into 0–3 vs. 4–6, no significant difference between coiling and clipping persists. This aligns with more recent critiques, such as that by Ganesh et al. [19], advocating for ordinal analysis of the mRS rather than binary endpoints, to better capture the clinical spectrum of outcomes in randomized stroke trials. These findings collectively highlight the importance of nuanced outcome assessment and suggest caution in overinterpreting trial results based solely on dichotomized disability scales. Against this backdrop, the conclusions drawn from the so far existing RCTs should be tailored on an individual patient level. Hence, the stratified comparison between clipping and coiling regarding aneurysm location is of importance.

### Functional outcome stratified by aneurysm location

In aSAH, AComA aneurysms frequently rupture at small sizes and are associated with complex anatomy such as A1 hypoplasia, which can increase surgical risk [20]. In our pooled analysis, functional outcomes did not differ significantly between clipping and coiling, supporting an individualized, anatomy-based approach in the acute setting.

#### Middle cerebral artery (MCA) aneurysms

MCA aneurysms after rupture are often surgically treated. A cohort study of 603 aSAH patients demonstrated that MCA aneurysms were associated with the best Glasgow Outcome Scores post-aSAH and exhibited the lowest incidence of DCI relative to other locations [21]. Our results showed no significant difference in 1-year functional outcome; however, the overall pooled effect across all locations favoring clipping in terms of complete occlusion was largely driven by the MCA subgroup.

#### Internal carotid artery (ICA) aneurysms

After rupture, ICA aneurysms—particularly at the ophthalmic segment or bifurcation—pose surgical challenges. Proximal control in the paraclinoid location after aSAH can be challenging and often necessitates cervical control of the ICA or tools for flow reduction. These features might contribute to our pooled results showing that functional outcomes in this location are better after coiling, likely reflecting the higher morbidity risk of microsurgical access in this location [7, 22].

#### Posterior circulation aneurysms

Aneurysms of the posterior circulation are associated with increased in-hospital mortality and greater incidence of acute hydrocephalus, which significantly influences the functional outcome after aSAH [21]. Their deep anatomical location and proximity to critical perforating vessels complicate surgical access, often making endovascular treatment the preferred option despite inherent technical challenges [23, 24]. This specific result may be influenced by the fact that posterior communicating artery aneurysms located at the internal carotid artery are frequently assigned to the posterior circulation with similar results for both treatment modalities, and by the underrepresentation of basilar and cerebellar artery aneurysms in prospective randomized trials [10].

### Aneurysm occlusion at 1-year after surgery

The present meta-analysis of complete aneurysm occlusion at 1-year after aSAH based on angiography shows that clipping is superior to coiling in 1889 patients from RCTs. This finding is consistent with previous reports demonstrating a 33% higher incidence of complete aneurysm closure compared to coiling [25]. Although endovascular techniques have advanced substantially, surgical clipping even remains the preferred treatment for MCA aneurysms in high-volume cerebrovascular centers in recent RCTs [5]. The aneurysm location-specific subgroup analysis for ACA, MCA, ICA, and posterior circulation shows that this effect is predominantly based on the superiority of clipping in MCA aneurysms. Rates of complete occlusion among ICA, ACA, and posterior circulation showed no difference between clipping and coiling. Clipping in MCA aneurysms enables the preservation of distal flow and minimizing the risk of perforator-related complications [24, 25]. Anatomically, MCA aneurysms often resemble complex bifurcation lesions rather than simple saccular sacs, frequently involving large necks and critical branch vessels. Their treatment requires precise reconstruction and careful microsurgical technique. [26, 27] Against this backdrop, clipping is the modality of choice for MCA. The ARETA [28] trial showed that approximately 20% of coiled aneurysms exhibit reopening or neck remnants, with about half of these requiring retreatment, and the CARAT [29] study linked the risk of early rebleeding to the extent of occlusion of the aneurysm emphasizing the goal of achieving complete occlusion while preventing treatment-associated complications [7].

### Limitations

Endovascular treatment for aSAH has evolved substantially, with modern coiling techniques and adjunctive devices differing markedly from those used in earlier RCTs. These advances have enabled more tailored and less invasive interventions, potentially improving safety and efficacy [30]. Although ISAT-2 [5], the most recent RCT, is included in our meta-analysis, subgroup analyses assessing the role of newer devices such as the Woven EndoBridge (WEB) device or stent-assisted techniques are not yet feasible or published. The included RCTs span more than 2 decades, during which both surgical and endovascular techniques have evolved considerably, potentially affecting the comparability of results. Device heterogeneity, particularly in the endovascular arm, introduces variability that may confound treatment effects. The relative under-representation of patients with posterior circulation aneurysms and high-grade aSAH limits the generalizability of our results to these subgroups. Although complete occlusion was not designated as the primary endpoint in the original randomized controlled trials (RCTs), it serves as the primary endpoint for the current meta-analysis. This methodological discrepancy may introduce a distribution bias in our secondary data analysis, as anatomical localization was not randomly distributed across the treatment cohorts in the included trials. Against this backdrop, the present meta-analysis includes subgroup analyses stratified by aneurysm location.

## Conclusions

In this meta-analysis of six RCTs on aSAH, coiling was associated with better 1-year functional outcomes when using mRS 0–2 as the cut-off, while clipping achieved superior complete occlusion rates—predominantly in MCA aneurysms. No mortality difference was observed, and outcomes varied by aneurysm location, underscoring the importance of individualized treatment selection. These findings support a location-specific approach in clinical decision-making, balancing functional prognosis with long-term occlusion durability.

## Supplementary Information

Below is the link to the electronic supplementary material.

Supplementary file1 (DOCX 198 KB)

## Data Availability

The data analyzed in this meta-analysis were derived from previously published studies and are available from the corresponding publications cited in this article. No new primary data were collected for this study.

## Abbreviations

aSAH: Aneurysmal subarachnoid hemorrhage
ACA: Anterior cerebral artery
ACom: Anterior communicating artery
CI: Confidence interval
ICA: Internal carotid artery
ISAT: International Subarachnoid Aneurysm Trial
MCA: Middle cerebral artery
mRS: Modified Rankin Scale
OR: Odds ratio
PRISMA: Preferred Reporting Items for Systematic Reviews and Meta-Analyses
RCT: Randomized controlled trial
WFNS: World Federation of Neurosurgical Societies
