# Supplementary material for: Efficacy and safety of endovascular coiling compared to microsurgical clipping in aneurysmal subarachnoid hemorrhage: a systematic review and meta-analysis of randomized trials
Source: J Neurol. 2026 Oct 8;273(10):650. doi: 10.1007/s00415-026-14150-z (PMC13649945; doi:10.1007/s00415-026-14150-z)
Supplement: Supplementary file 1 [file 415_2026_14150_MOESM1_ESM.docx]

**SUPPLEMENTAL DIGITAL CONTENT LEGENDS**

**Supplemental Digital Content 1. Supplemental figure 1 –** Prisma Flow-Chart

**Identification of studies via databases and registers**

**Records removed *before screening*:**

Non-clinical studies, experimental studies, letters, case reports, technical notes (n = 1269)

**Records identified from:**

Pubmed, Cochrane, Embase (n = 1368)

**Identification**

Non-controlled trials excluded

(n = 46 )

Records screened:

(n = 99)

**Screening**

Reports excluded:

Pharmacological study, endovascular/microsurgical treatment solely

Abstracts screened:

(n = 53)

Studies included for outcome analysis mRS 0-2 vs. mRS 3-6 (**n = 6**)

Studies included for outcome analysis mRS 0-3 vs. mRS 4-6 (**n = 3**)

Studies included for outcome analysis based on aneurysm localization (**n = 2**)

Studies included for outcome analysis based on WFNS grage (**n=2**)

Studies included for mortality analysis (**n = 6**)

Studies included for complete occlusion analysis (**n = 4**)

Studies included for complete occlusion analysis based on aneurysm location (**n = 2**)

**Included**

**Supplemental Digital Content 2. Supplemental figure 2 –** A: Forest plot displaying odds ratio (OR) and 95% confidence interval (CI) estimates for favorable clinical outcome (mRS 0–3) from randomized controlled trials comparing endovascular coiling versus surgical clipping for intracranial aneurysms. Squares indicate study-specific ORs, with square size reflecting study weight. The diamond represents the pooled OR from the random-effects model. B: Funnel plot assessing publication bias for favorable clinical outcome (mRS 0–3). The plot shows a symmetrical distribution of studies, indicating no evidence of publication bias.

**Supplemental Digital Content 3. Supplemental Figure 3 –** A: Forest plot displaying odds ratio (OR) and 95% confidence interval (CI) estimates for favorable outcome by patients with WFNS ≤ 3. Squares represent individual study ORs, with square size proportional to the study’s weight (inversely related to CI width). The diamond represents the pooled OR, demonstrating the overall effect estimate from all included studies. B: Funnel plot assessing publication bias. The distribution of included studies is symmetrical, indicating no relevant publication bias.

**Supplemental Digital Content 2. Supplemental Figure 4** **–** A: Forest plot displaying odds ratio (OR) and 95% confidence interval (CI) estimates for favorable outcome by patients with WFNS > 3. Squares represent individual study ORs, with square size proportional to the study’s weight (inversely related to CI width). The diamond represents the pooled OR, demonstrating the overall effect estimate from all included studies. B: Funnel plot assessing publication bias. The distribution of included studies is symmetrical, indicating no relevant publication bias.

**Supplemental Digital Content 2. Supplemental Figure 5** **–** A: Forest plot displaying odds ratio (OR) and 95% confidence interval (CI) estimates for mortality from randomized controlled trials comparing endovascular coiling versus surgical clipping for intracranial aneurysms. Squares indicate study-specific ORs, with square size reflecting study weight. The diamond represents the pooled OR from the random-effects model. B: Funnel plot assessing publication bias for mortality. The plot shows a symmetrical distribution of studies, indicating no evidence of publication bias.
